# Burnout Syndrome in Selectable Athletes for the Brazilian Handball Team—Children Category

**DOI:** 10.3390/ijerph20043692

**Published:** 2023-02-19

**Authors:** Max dos Santos-Afonso, Luciano Garcia Lourenção, Marla dos Santos Afonso, Mirelle de Oliveira Saes, Fernando Braga dos Santos, José Gustavo Monteiro Penha, Daniela Menezes Galvão, Francisco Rosemiro Guimarães Ximenes Neto, Natalia Sperli Geraldes Marin dos Santos Sasaki, Maria de Lourdes Sperli Geraldes Santos, Flávio Adriano Borges, Jacqueline Flores de Oliveira, Sidiane Teixeira Rodrigues, Eliel de Oliveira Bandeira, Francisco Cavalcante de Alcantara, Carlos Leonardo Figueiredo Cunha, Francielle Garcia da Silva, Messias Lemos, Abelardo de Oliveira Soares Junior, Fernanda Burlani Neves

**Affiliations:** 1 Postgraduate Program in Health Sciences, Federal University of Rio Grande, Rio Grande 96201-900, Rio Grande do Sul, Brazil; 2 Nursing School, Federal University of Rio Grande, Rio Grande 96201-900, Rio Grande do Sul, Brazil; 3 Health Sciences Center, Vale do Acaraú State University, Sobral 62042-280, Ceará, Brazil; 4 School of Medicine of São José do Rio Preto, São José do Rio Preto 15090-000, São Paulo, Brazil; 5 Nursing Department, Federal University of São Carlos, São Carlos 13565-905, São Paulo, Brazil; 6 Public Health Department, Federal University of Maranhão, São Luís 65020-070, Maranhão, Brazil; 7 Nursing Department, Federal University of Santa Catarina, Florianópolis 88040-900, Santa Catarina, Brazil; 8 Institute of Biology, Federal University of Pelotas, Pelotas 96001-970, Rio Grande do Sul, Brazil

**Keywords:** burnout, professional, athletes, youth sports, Brazilian handball team

## Abstract

To investigate the presence of burnout syndrome in child athlete tryouts for the Brazilian Handball Team, before and after the National Development and Technical Improvement Camp is of great interest. A correlational study, with longitudinal design of the before-and-after type, carried out with 64 male athletes in the children’s category, immersed in the National Camp for Development and Improvement of Handball Technique, in the municipality of São Bernardo do Campo, São Paulo, Brazil, in December 2018. To evaluate burnout syndrome, we used the Athlete Burnout Questionnaire (ABQ). There was a statistically significant increase of the mean scores for burnout and dimensions (Physical and Emotional Exhaustion = 1.5 to 1.6; *p*-value < 0.001; Reduced Sense of Accomplishment = 2.7 to 2.9; *p*-value < 0.001; Sports Devaluation = 1.4 to 1.6; *p*-value < 0.001; and General Burnout = 1.9 to 2.0; *p*-value < 0.001). The athletes selected for the national team had lower mean scores for general burnout and dimensions (Physical and Emotional Exhaustion = 1.5; Reduced Sense of Accomplishment = 2.7; Sports Devaluation = 1.5; General Burnout = 1.9). The National Camp for Development and Technical Improvement can have a negative impact on the mental health of athletes. This event is important to select the competitors with greater ability to face the pressure and adversities present in the sport environment.

## 1. Introduction

The environment of sports competitions requires an intense load of physical training, which generates pain, wear and tear, and fatigue. In addition to physical commitment, there are feelings of high responsibility and many demands from family members, colleagues, and coaches for a good performance [1]. When athletes do not achieve the desired performance, either physically or psychologically, frustrations and emotional impacts occur, which increase the level of stress [2]. 

The search for victory and overcoming opponents, increasingly present during competitions, causes athletes to reach their emotional limit, culminating in mental exhaustion that characterizes burnout syndrome [3]. Among elite athletes, mental exhaustion resulting from the stress generated by high performance sports has become a concern after debates emerging from reports and worrying experiences of some professionals, for example Simone Biles in the Tokyo Olympics (2021) [4].

In Brazil, the Brazilian Handball Confederation annually organizes the Development and Technical Improvement Camp. This event has the objective of seeking new talents who can represent Brazilian handball in the future. The event has regional stages that precede the national stage. In the Regional Camps, the Brazilian Handball Confederation sends a qualified technician who is responsible for standardizing the handball techniques applied in Brazil and indicating the best talents to participate in the National Camp [5]. These camps aims to train the players so that they can pursue a professional career, as well as transmit the concepts of the camp’s entity and its representatives. Many athletes who today are part of youth or adult national teams went through camps similar to this one.

However, it is believed that National Camp for Development and Technical Improvement of Handball is a competitive environment which can favor the onset of burnout syndrome in the competitors. Confirming this hypothesis is important, as in the sports environment, enduring stress can turn into burnout, causing frustrations and aggravating clinical cases [6]. Furthermore, the chronic stress in childhood and adolescence can lead to problems in adulthood, such as depression, anxiety disorders, low self-esteem, and increased risk for suicide [7]. 

Burnout syndrome was first defined in the early 1970s as a behavioral pattern that included progressive loss of energy, demotivation, lack of all interest in work reaching exhaustion. Four and a half decades later, the syndrome has become a popular topic in a variety of social contexts, including among athletes, coaches, and technical committees in the sports field [8]. In 2020, it was defined by the World Health Organization as an occupational disease, being considered a work-related phenomenon [9].

In the sports context, burnout has been referred to as a multidimensional syndrome caused by a complex interaction between multiple factors of intense demands, inadequate recovery, and frustrations from unmet expectations. As a consequence, athletes lose motivation, pleasure, satisfaction, and interest in playing sports. As a result of this framework, they demonstrate a perception that the physical and mental efforts requested in training and competitions do not compensate for the benefits eventually accumulated [10].

Thus, the evaluation of the motivational behavior of athletes can help in improving sports training and in reducing the number of abandonments of sports practice [3], because the injuries and overloads that involve the sports world are caused mainly by the rigidity of training and competitions [11]. 

In this context, if an elite athlete abandons his or her sport due to physical, mental, or emotional exhaustion, one realizes a gigantic shock to talent development, as other athletes may experience the same suffering. Thus, investigations about the link between burnout and abandonment of sport are considered important for understanding the impact of physical and emotional exhaustion on individual development and loss of talent [12]. 

Analyzing the presence of mental exhaustion in the sport context is essential to improving the quality of life and performance of athletes, especially younger athletes, with the possibility of a promising career. In this sense, to explore burnout syndrome in child athletes is relevant as it favors the planning of prevention actions and health promotion in the sports base.

A study with 97 Brazilian adolescents, handball athletes of both sexes, identified moderate levels of stress in the pre-competition period [13]. Similarly, nearly 70% of a sample of more than one hundred child swimmers in northern Brazil indicated that they did not achieve their goals or that their sport performance fell below expectations due to overload in the sport [14]. In another study of 368 college athletes, anxiety had a significant correlation with mental exhaustion, evidencing that the more anxious athletes felt, the less personal fulfillment they felt [15]. 

A study conducted in Spain with tennis athletes aged 8 to 15 years indicated that 32% had a high risk of suffering from burnout syndrome [16]. In another study of 442 Spanish sportsmen and women, only 12.4% were at high risk of burnout syndrome [8]. Similar results were obtained in a study of 397 Spanish athletes, from various sports, aged 13 to 14 years [17]. 

A meta-analysis of a review article suggests that, over the past two decades, there has been a substantial increase in burnout symptoms among athletes [18], reinforcing other international studies that point out that fatigue and mental exhaustion affect the performance of competitors of this modality [19,20,21,22].

In this context, it is essential to investigate the presence of stress and burnout among high performance athletes, to assist them in the development of an effective therapeutic project in the follow-up of their career. This project is based on mental training techniques and encompasses a series of practices that strengthen the competitor’s mental abilities, such as focus, emotional control, and resilience. By means of repetitive exercises, the techniques organize emotional, cognitive, and motivational processes, including relaxation, goal setting, and internal dialogue [23].

Therefore, this study aimed to investigate the presence of burnout syndrome in child athletes selectable for the Brazilian Handball Team, before and after the National Development and Technical Improvement Camp.

## 2. Materials and Methods

### 2.1. Type of Study

To investigate the presence of burnout syndrome, an observational, descriptive, and correlational study with longitudinal design of the before-and-after type was conducted in 2018 with male athletes of the children’s category who were immersed in the National Camp for Development and Improvement of Handball Technique in the municipality of São Bernardo do Campo, São Paulo, Brazil.

### 2.2. Sample and Participants

The study population consisted of 64 male athletes from the children’s category (12 to 14 years old) who participated in the National Camp for Development and Improvement of Handball Technique, aiming for a place on the Brazilian Handball Team. Those who agreed to participate were included in the study after their parents’ consent. 

The athletes who participate in the Development and Technical Improvement Camps have practical and theoretical classes, giving them the opportunity to learn more, put their knowledge into practice, and show their talents. In addition, the activities also count on the presence of coaches, local teachers, and teams of health professionals (doctors, physical educators, and physiotherapists) who seek to learn more about the guidelines of the work done with the athletes and to disseminate them in their working environment. The aim is, therefore, to standardize the way handball is played across Brazil, as well as to discover talent that can bring the renewal of the Brazilian team.

After regional triages carried out with more than two thousand athletes all over Brazil, 64 young athletes reached the national phase. These athletes are evaluated by more than three hundred coaches and teachers, and the candidates with the best results are selected. The State Federations also contributes to the line-up by indicating players who perform well during the season.

All 64 athletes participated in the study. As demonstrated in Table 1, athletes were aged between 13 (25.0%) and 14 (75.0%) years old; 37 (57.8%) considered themselves white and 51 (79.7%) had no paid activity. Although most of the athletes had no information about their family income, it was observed that there was a predominance of socio-economic classes C, D, and E (31.2%), classes whose family income was lower than 2580 dollars (BRL 10,000.00). 

Regarding schooling, most athletes (51.6%) were in the ninth grade, and 28 (43.8%) had always studied in private schools (with or without scholarships). A significant number of athletes came from states in the southeast region (40.6%), and 16 (25.0%) were from the state of São Paulo; 48 (75.0%) lived in rented houses and, for 41 (64.1%), the father and mother were responsible for the family support. 

Forty-one (64.1%) athletes practiced handball at school and/or clubs in their cities of origin, without receiving a salary; 23 (35.9%) athletes trained from six to nine hours a week and 20 (31.3%) trained from 10 to 20 h a week.

### 2.3. Procedures, Measurements, Variables and Outcome

For data collection, two instruments were used. Firstly, a questionnaire developed by the researchers, containing information about the sociodemographic and professional profile of the athletes: identification variables (date of birth, place of birth, city of origin, state of origin, has a paid job, school grade, responsibility for the supporting family); ethnic-racial identity; housing situation (owned or rented); educational context (type of school and if they had a scholarship); sports context (when and how much they practice the handball activity); family group data (bond of the people who live with the athlete, profession of the people who live with the athlete, family income). Secondly, the Athlete Burnout Questionnaire (ABQ), was translated and confirmed in the Portuguese language by Pires, Brandão, and Silva [24]. This questionnaire verifies the athletes’ perception about their sport performance and how they feel in relation to professional achievement [24,25].

The ABQ consists of 15 questions, structured in a Likert scale of five points: five questions to measure the sub-area’s ‘physical and emotional exhaustion’, five to measure the sub-area ‘sport devaluation’, and five to measure the sub-area ‘reduced sense of sport accomplishment’. The number 1 means ‘I hardly ever feel this way’, and 5, ‘I feel this way most of the time’. The intermediate frequencies are “rarely” (2), “sometimes” (3), and “often” (4). The instrument does not have a specific score. The classification is made based on a continuum that starts with a minimum of 15 points and reaches a maximum of 75 points [26].

The results are obtained from the arithmetic mean of the answers given to the five items corresponding to each burnout dimension. The general burnout value is calculated by the arithmetic mean of the 15 items of the instrument. The interpretation of the scores is done by using the variation of the frequency of feelings. This means that if an athlete obtains a mean score of 2.50 for the dimension physical and emotional exhaustion, it is considered that this individual presents feeling related to this dimension with a frequency from rarely to sometimes [27].

Data were collected at two different moments: upon arrival of the athletes at the National Handball Development Center Prof. José Maria Passos, in the city of São Bernardo do Campo, São Paulo, and upon departure of the athletes, after 10 days of immersion in the National Camp for Development and Improvement of Handball Technique. 

In the first moment, the 64 athletes were informed about the study’s objective and methodology and were asked to sign the Free and Informed Consent Form. Soon after, they answered the data collection instruments. After 10 days of immersion, at the end of the National Handball Development and Technique Improvement Camp, the 64 athletes answered the Athlete Burnout Questionnaire (ABQ) again.

In order not to influence the perception of the young athletes, the results of the first collection were not published before the second collection.

### 2.4. Statistical Analysis

The data were analyzed using the Statistical Package for Social Sciences (SPSS), version 20.0 (IBM: New York, USA). The Kolmogorov–Smirnov normality test was used to verify data distribution. The internal consistency indicator Cronbach’s Alpha was used to verify the reliability of the measures of the ABQ constructs. Then, the data were treated with descriptive statistics (frequency and percentage, mean, and standard deviation) and inferential statistics (comparison of mean scores). 

To compare the mean scores obtained in the ABQ constructs, on arrival and at the end of the camp, the *t*-test was used. To compare the mean scores of the ABQ constructs with the sociodemographic variables, the *t*-test for two mean scores or Analysis of Variance (ANOVA) for three or more mean scores was used, considering a significance level of 5% (*p* ≤ 0.05).

### 2.5. Ethical Considerations

Ethical approval regarding this study was obtained from the institutional ethics committee (decision: 3,079,959—December 13, 2018; CAAE: 04111418.0.0000.5415). All the participants in this study were only included after informed consent had been obtained from them and its responsible parties. All procedures performed in this study were in accordance with the ethical standards of the institutional research committee and with the comparable ethical standards outlined in the Declaration of Helsinki.

## 3. Results

The analysis of general burnout levels and dimensions upon arrival and at the end of the National Development and Technical Improvement Camp, showed that the child athletes had low mean scores at both times. However, there was a statistically significant increase of the values for general burnout and dimensions at the end of the camp compared to the initial evaluation. According to the ABQ, the mean scores upon arrival and at the end of the camp, were from “almost never” to “rarely” for the dimension Physical and Emotional Exhaustion and Sports Devaluation, and for general burnout; and from “rarely” to “sometimes” for the dimension Reduced Sense of Accomplishment, as shown in Table 2.

Table 3 shows the distribution of the mean scores of the ABQ dimensions and general burnout, according to the athletes’ socio-demographic and professional variables. Although there were no statistically significant differences between values observed, a tendency towards lower levels for all dimensions and general burnout was noted among Black athletes and those who do not work; higher levels of general burnout and dimensions Reduced Sense of Accomplishment and Sport Devaluation were found among athletes who have always studied in public schools. 

Athletes from the northeast tend to have lower mean values for general burnout and dimensions of Reduced Sense of Accomplishment and Physical and Emotional Exhaustion, while the athletes from the southern region presented higher mean values for general burnout and dimensions of Reduced Sense of Accomplishment and Physical and Emotional Exhaustion. 

Athletes who train in the city school or club and do not receive a salary presented higher mean scores for general burnout and dimensions of Reduced Sense of Accomplishment, and Physical and Emotional Exhaustion, while the athletes who train in a sports club and receive a salary presented lower mean scores for general burnout and dimensions of Reduced Sense of Accomplishment, and Physical and Emotional Exhaustion. 

The athletes with longer weekly training time presented lower levels of Physical and Emotional Exhaustion, Reduced Sense of Accomplishment, and general burnout. On the other hand, they presented a higher mean score for Sport Devaluation.

The comparison of burnout levels between selected and non-selected athletes pointed out that the twenty athletes who were selected for the national team at the end of the camp had lower mean scores for all ABQ dimensions and general burnout (Table 4).

## 4. Discussion

The results of the study show that almost 10% of the athletes are enrolled in the sixth and seventh grades of elementary school and, therefore, are behind in relation to their age. This delay in school development can directly influence the athlete’s performance, since in order to participate in the regional and national camps it is necessary to be enrolled in a public or private educational institution and to present good school performance. According to the United Nations Children’s Fund (UNICEF), the delay in school development is a cumulative phenomenon that starts in the early years of elementary school and continues throughout the school career. This age/grade distortion intensifies in the final years of elementary school, making many students unable to complete the school journey with quality and at the expected age, a fact that can cause the loss of potential talents for Brazilian handball [28].

According to the Copenhagen Consensus Conference, the practice of physical activity is beneficial for the age group in question, bringing benefits to the health and quality of life of these adolescents and favoring cognitive development [29]. However, although these are athletes who aim to integrate the Brazilian male children’s handball team, it is clear that the amount of training may be below what is necessary for a high level of sports performance [30].

The statistically significant increase in the mean scores for the ABQ dimensions and general burnout, obtained by the athletes after the camp, confirms the hypothesis that the Development and Technical Improvement Camp, held annually by the Brazilian Handball Confederation is a competitive environment which can favor the onset of Burnout Syndrome in the competitors. Corroborating these findings, Fernández-Rio, Cecchini, and Méndez-Giménez [31] observed that the levels of burnout in handball players increased after an intense training session. For the authors, high intensity training can produce negative effects on the motivation and self-determination of athletes.

Although the range of mean pre and post-camp mean scores presented low variation and show low risk of occurrence of Burnout during short period competitions, it is believed that throughout a career of intense training and competitions, the athletes will be more susceptible to the development of the syndrome. These results may have occurred because they are child athletes who develop their skills in other activities and have a less intense period of training and competitions, unlike high performance professional athletes, who dedicate themselves exclusively to their sport [32]. In this context, a study with professional athletes from the volleyball league indicates an increase in the perception of burnout by the athletes as the cycle of competitions advances [33], reinforcing that burnout is a chronic process [34]. 

The differences in the range of pre and post-camp mean scores observed in this study may be related to the existence of risk factors, such as overtraining, injuries and bruises, poor performance, impaired sleep, anxiety, and low social support, which may interfere with the mental health of athletes, especially elite athletes, potentiating the onset and severity of specific mental health symptoms [35,36,37,38,39,40]. Furthermore, the risk of stress increases when athletes lack sufficient coping resources to deal with high emotional demands. If stress is prolonged, it can lead to burnout [33]. Therefore, it is important that those responsible for the camp activities create a welcoming environment where athletes feel integrated and comfortable to share their emotions and ask for social support when needed [41,42,43].

In contrast, corroborating the results of this study, Spanish athletes of diverse sport modalities, aged 13 years and older, presented low scores for the ABQ dimensions and general burnout. However, the authors evidenced that, despite the low scores, there is a relationship between stress, burnout, and depression in professional athletes [44]. Moreover, there is a tendency for athletes of team sports to present a lower perception of feelings inherent to the burnout syndrome due to the greater number of interpersonal relationships and the division of functions experienced by these athletes in the competitive environment [45].

However, in recent years, the COVID-19 pandemic has restricted social contact and interpersonal relationships among athletes, causing a negative impact on the mental health of athletes [46], especially those competing in individual sports [47]. 

The higher mean score obtained by athletes in the dimension Reduced Sense of Achievement may be an indication of low satisfaction with their skills, dexterity, and accomplishments, stemming from the fact that they are far from reaching the peak of their professional careers. Besides, pressure from family members and coaches may compromise the athlete’s performance, decreasing the success in competitions and increasing the feeling of dissatisfaction [32]. These results can still be aggravated by the financial difficulties experienced by the athletes, especially those coming from medium and low socioeconomic classes, as well as by the deficit of resources destined to the Brazilian sport, since the sportive gear has a high cost and the maintenance of an athlete of high performance in sport, mainly in the infantile categories (13 and 14 years), cadet (15 and 16 years) and juvenile (17 and 18 years) demands high investment [48,49,50].

Regarding the propensity of athletes who have always studied in public schools to present higher levels of General Burnout, Reduced Sense of Achievement and Sport Devaluation, it is noteworthy that 20% of the students enrolled in Brazilian public schools present age above the recommended for the series [51], a fact that may aggravate the emotional distress suffered by the athletes. This argument is reinforced by the fact that athletes who study in private schools tended to have lower averages for all ABQ dimensions and general burnout. 

On the other hand, athletes who train in the city school or club and do not receive a salary tended to present higher mean scores for general burnout and dimensions, such as Reduced Sense of Accomplishment and Physical and Emotional Exhaustion, corroborating the international literature [6]. As sports programs have become increasingly expensive, adolescents with this profile are more susceptible to the negative impacts of the physical, mental and emotional overload inherent to the practice of competitive sports [52].

Nevertheless, the investment in sports by Brazilian federal government is increasingly smaller. In the proposal of the Annual Budget Law of 2020, the Brazilian government predicted an investment of 220 million Reais (BLR 220,000,000.00) for sport, which represents a cut of almost 50% in relation to the previous year’s budget, a situation that worsened in the following years [53,54].

Moreover, not all athletes in the children’s category are objects of this study and meet the age requirement to receive the Athlete Grant, a financial incentive program of the federal government, which aims to contribute to the training of athletes with the potential to represent Brazil in the most diverse sports modalities, granted to athletes from 14 years old [50], favoring negative impacts on the mental health of these athletes.

Research indicates that parents feel stress due to the need to allocate resources for children within the family unit, whether they are athletes or not [55]. Although many risk factors such as socioeconomic status or relationship with parents are not investigated [56], it was observed in this study that 64.0% of athletes have their parents as family breadwinners, i.e., they have family support, even when they belong to the lower socioeconomic classes. 

The supportive relationship with parents and coaches is essential for the athlete’s well-being in the child development years, and it is at this stage of career development that mental health problems become obvious and need special attention from the athletes themselves, coaches, and parents [57]. Coaches, in particular, play an important role in this development process, as they will provide support for athletes for many years, especially for the youth athletes [58].

The fact that the athletes selected for the national team present a tendency to have lower levels of mental and emotional exhaustion is a positive factor, which demonstrates a greater capacity of these athletes to face the pressure and adversities present in this highly competitive and stressful environment, which increases the risk of physical and emotional exhaustion, performance anxiety, reduced satisfaction with the sport, and a decrease in the athletes’ psychological well-being [59,60].

This enhanced emotional performance, which, even if subtle, is important, since mental fatigue impairs athlete performance in some sport-specific tasks. At the elite competition level, where results are determined by very small margins, reducing the impact of mental fatigue on athlete performance is an important differentiator [61].

Finally, it is emphasized that the mental health of elite athletes is highly vulnerable, due to pressures and stresses that are considered risk factors for the development of mental disorders and burnout, which can lead to loss of performance and early abandonment of the athletic career. Therefore, it is essential to identify the presence of mental and emotional exhaustion among athletes in order to target the implementation of stress reduction techniques in an attempt to improve competitive performance [12,62,63]. 

This study presents some limitations, such as the small number of participants, the evaluation of only one sport category, inclusion of only subjects in the male modality, and the period of training and preparation evaluated. On the other hand, 64 youngsters who went through regional triages were evaluated, with more than two thousand athletes all over Brazil. However, the results contribute important knowledge about the preparation and selection of athletes for the Brazilian men’s handball team, favoring the discussion about the impact of the activities of the National Development and Technical Improvement Camp on the health of these adolescents. Furthermore, it allows the reflection on possible negative impacts caused to athletes during Regional Camps, which precede the national stage, enabling the creation and implementation of actions that seek to minimize the negative impacts and enhance the development of future athletes of elite handball.

This study can be considered as a basis to facilitate the development of athlete health support services throughout the athlete’s career in handball and in other team sports, especially in the national teams, aiming at health promotion and prevention, a healthy sport involvement, good performance, personal and professional development, and good mental health and well-being. 

It is also evident the importance of the performance of health professionals, such as physiotherapists, psychologists, nurses, nutritionists, doctors, and physical educators in the process of athletes’ formation, especially in the base categories such as children, cadets, and teenagers.

## 5. Conclusions

The results of the study showed that the National Camp for Development and Technical Improvement, held annually by the Brazilian Handball Confederation can have a negative impact on the mental health of athletes. At the end of the camp, the athletes presented statistically higher means for all subscales of the ABQ and for total burnout in relation to answers obtained upon arrival at the camp. 

These results provide support for the discussion about possible negative impacts caused to the athletes during the Regional Camps, which precede the national stage, enabling the creation and implementation of actions that seek to minimize the negative impacts and enhance the development of future athletes of handball elite.

In addition, the study points out that the National Camp for Development and Technical Improvement is important to select the competitors with greater ability to face the pressure and adversities present in the sport environment.

## Figures and Tables

**Table 1:** Sociodemographic and professional characteristics of selectable athletes for the Brazilian handball team—children category.

Variables	*n*	%
Age		
13 years old	16	25.0
14 years old	48	75.0
Ethnic-Racial Identity		
White	37	57.8
Brown	20	31.3
Black	7	10.9
Has a paid job		
Yes	13	20.3
No	51	79.7
Monthly Gross Family Income *		
Class A [Above USD 4836.13 or BRL 18,740.01]	1	1.6
Class B [USD 2418.06 or BRL 9370.01 to USD 4836.12 or BRL 18,740.00]	6	9.4
Class C [USD 967.22 or BRL 3748.01 to USD 2418.06 or BRL 9370.00]	9	14.1
Class D [USD 483.61 or BRL 1874.01 to USD 967.22 or BRL 3748.00]	6	9.4
Class E [Up to USD 483.61 or BRL 1874.00]	5	7.8
Did not know how to answer	37	57.8
Grade that you are enrolled in		
Sixth grade	1	1.6
Seventh grade	5	7.8
Eighth grade	25	39.1
Ninth grade	33	51.6
Educational Context		
Studying in a public school	25	39.1
Study in private school	28	43.8
Studies in public and private school	11	17.2
Brazilian Region of Origin		
Southeast	26	40.6
South	20	31.3
Center-West	10	15.6
Northeast	6	9.4
North	2	3.1
Housing Situation		
Rented	16	25.0
Owned	48	75.0
Responsible for family support		
Father and Mother	41	64.1
Father	12	18.8
Mother	6	9.4
Mother and grandmother	1	1.6
My parents and I help	4	6.3
Where you practice handball		
School/City Club—No salary	41	64.1
City School/Club—With Salary	5	7.8
City Club—No salary	13	20.3
Sport Club—With Salary	5	7.8
Hours of handball practice per week		
Up to five hours	10	15.6
From six to nine hours	23	35.9
From 10 to 20 h	20	31.3
More than 20 h	1	1.6
Did not know how to answer	10	15.6

* Classification from the Brazilian Institute of Geography and Statistics (IBGE), 2019. USD 1 = BRL 3.8750.

**Table 2:** Distribution of the pre and post competition mean scores obtained by the athletes, according to ABQ dimensions and general burnout.

	Pre-Competition	Post-Competition	*p*-Value
	Mean (±sd)	Mean (±sd)	(*t* Test)
Physical and Emotional Exhaustion	1.5 (±0.4)	1.6 (±0.5)	<0.001
Reduced Sense of Accomplishment	2.7 (±0.5)	2.9 (±0.5)	<0.001
Sports Devaluation	1.4 (±0.5)	1.6 (±0.6)	<0.001
General Burnout	1.9 (±0.3)	2.0 (±0.4)	<0.001

sd: standard deviation.

**Table 3:** Distribution of the mean scores for ABQ dimensions and general burnout, according to athletes’ sociodemographic and professional variables.

Variables	PEE	RSA	SDE	General Burnout
Ethnic-Racial Identity				
White	1.7 (±0.6)	2.9 (±0.6)	1.5 (±0.7)	2.0 (±0.4)
Brown	1.6 (±0.6)	2.9 (±0.4)	1.7 (±0.6)	2.0 (±0.4)
Black	1.4 (±0.4)	2.5 (±0.2)	1.4 (±0.3)	1.8 (±0.2)
*p*-value **	0.269	0.144	0.535	0.247
Has a paid job				
Yes	1.4 (±0.4)	2.8 (±0.8)	1.4 (±0.5)	1.9 (±0.4)
No	1.7 (±0.6)	2.9 (±0.4)	1.6 (±0.7)	2.0 (±0.4)
*p*-value *	0.055	0.646	0.253	0.137
Monthly Gross Family Income *				
Class A	2.0 (±0.0)	2.6 (±0.0)	1.4 (±0.0)	2.0 (±0.0)
Class B	1.8 (±0.6)	2.9 (±0.5)	1.7 (±0.5)	2.1 (±0.5)
Class C	1.6 (±0.5)	3.0 (±0.8)	1.5 (±0.4)	2.0 (±0.4)
Class D	1.5 (±0.8)	2.8 (±0.4)	1.3 (±0.3)	1.8 (±0.4)
Class E	1.6 (±0.8)	2.8 (±0.3)	1.8 (±0.7)	2.1 (±0.5)
*p*-value **	0.884	0.935	0.845	0.896
Educational Context				
Studying in a public school	1.6 (±0.6)	2.9 (±0.6)	1.6 (±0.6)	2.1 (±0.4)
Study in private school	1.6 (±0.5)	2.7 (±0.4)	1.5 (±0.8)	2.0 (±0.5)
Studies in public and private school	1.7 (±0.5)	2.9 (±0.5)	1.4 (±0.4)	2.0 (±0.3)
*p*-value **	0.701	0.264	0.509	0.655
Brazilian Region of Origin				
Southeast	1.6 (0.5)	2.7 (0.4)	1.4 (0.4)	1.9 (0.4)
South	1.8 (0.6)	3.1 (0.6)	1.6 (0.8)	2.2 (0.5)
Center-West	1.7 (0.6)	2.9 (0.4)	1.9 (0.6)	2.2 (0.3)
Northeast	1.2 (0.0)	2.9 (0.4)	1.0 (0.0)	1.7 (0.1)
North	1.3 (0.3)	2.7 (0.3)	1.5 (0.5)	1.8 (0.3)
*p*-value **	0.281	0.059	0.215	0.060
When you practice Handball				
School/City Club—No salary	1.6 (0.6)	3.0 (0.5)	1.6 (0.7)	2.1 (0.4)
City School/Club—With Salary	1.3 (0.3)	2.8 (0.5)	1.2 (0.4)	1.8 (0.3)
City Club—No salary	1.8 (0.5)	2.7 (0.3)	1.5 (0.4)	2.0 (0.2)
Sport Club—With Salary	1.3 (0.5)	2.6 (0.4)	1.5 (0.7)	1.8 (0.4)
*p*-value **	0.214	0.157	0.545	0.246
How many hours do you practice Handball per week?				
Up to five hours	1.6 (0.4)	2.8 (0.4)	1.5 (0.6)	2.0 (0.3)
From six to nine hours	1.7 (0.6)	2.8 (0.4)	1.7 (0.8)	2.0 (0.5)
From 10 to 20 h	1.6 (0.5)	2.9 (0.4)	1.4 (0.4)	2.0 (0.3)
More than 20 h	1.2 (0.0)	2.0 (0.0)	2.2 (0.0)	1.8 (0.0)
*p*-value **	0.870	0.289	0.656	0.854

PEE = Physical and Emotional Exhaustion. RSA = Reduced Sense of Achievement. SDE = Sport Devaluation. * *t*-test. ** Variance Analysis (ANOVA).

**Table 4:** Distribution of the mean scores obtained by the athletes selected and not selected for the Brazilian handball team, according to the ABQ dimensions and general burnout.

	Not Selected	Selected	*p*-Value
	Mean (±sd)	Mean (±sd)	(*t* Test)
Physical and Emotional Exhaustion	1.7 (±0.6)	1.5 (±0.5)	0.566
Reduced Sense of Accomplishment	2.9 (±0.5)	2.7 (±0.4)	0.634
Sports Devaluation	1.6 (±0.7)	1.5 (±0.43)	0.273
General Burnout	2.0 (±0.4)	1.9 (±0.4)	0.568

sd: standard deviation.

## Data Availability

The datasets generated during the current study are not publicly available but are available from the corresponding author on reasonable request.
